# Traveling With Cancer: A Guide for Oncologists in the Modern World

**DOI:** 10.1200/JGO.19.00029

**Published:** 2019-07-10

**Authors:** Sharon Heng, Brett Hughes, Michael Hibbert, Mustafa Khasraw, Zarnie Lwin

**Affiliations:** ^1^ Monserrat North Lakes Day Hospital, North Lakes, QLD, Australia; ^2^ Royal Brisbane and Women’s Hospital, Herston, QLD, Australia; ^3^ Royal North Shore Hospital, St Leonards, NSW, Australia

## Abstract

**PURPOSE:**

Travel for patients with cancer has become more achievable because of gains in quality of life and overall survival. The risk assessment of these patients is complex, and there is a paucity of data to which clinicians can refer. We present the challenges of traveling with cancer and a review of the literature.

**METHODS:**

A review using Preferred Reporting Items for Systematic Reviews and Meta-Analyses guidelines was performed. A search using the terms ”cancer,” “advanced cancer,” ”metastases,” “brain edema,” “lymphoedema,” “pneumothorax,” ”pleural effusion,” “pericardial effusion,” pneumonitis,” “hypoxia,” “end-of-life,” and “shunt,” combined with “flying” and “air travel,” was conducted. The PubMed and Cochrane databases were searched for English-language studies up to December 2018. Studies, case reports, or guidelines referring to travel in the context of adult patients with malignancies were included. A total of 745 published articles were identified; 16 studies were included. An inclusive approach to data extraction was used.

**RESULTS:**

There were no specific criteria to deem a patient with cancer fit to travel. Neurologic, respiratory, and cardiac implications, and time from recent surgery or procedure need to be considered There was a lack of high-quality studies to inform decisions, but the British Thoracic Society and Aerospace Medical Association Medical Guidelines included recommendations for fitness to fly for patients with cancer.

**CONCLUSION:**

In the absence of large prospective studies, individual fitness to travel should be assessed on a case-by-case basis, bearing in mind that maximizing a patient’s ability to safely travel is an important goal for many individuals with cancer.

## INTRODUCTION

In 2018, global air traffic passenger revenues increased to $561 billion (an increase of 7.6%) compared with 2017.^1^ The growing number of people traveling by air has conspicuously made inflight medical emergencies more common.^2,3^ Traveling in general, and especially airplane travel, is a major source of stress with health risks, particularly in the context of a preexisting illness such as cancer.^4,5^

Whether it is to visit overseas relatives or friends, tick off special destinations of interest on a bucket list, or seek out therapies not available in their own countries, patients with cancer often contemplate travel. The past decade has seen a changing landscape in cancer survivorship and lifestyle for patients and carers alike.^6^ These improvements are the result of earlier detection and new effective therapies. Small-molecule oral targeted agents, immuno-oncology, and targeted radiation techniques are reshaping cancer care throughout the entire disease trajectory. Newer therapies not only permit better performance status but also afford better progression-free and overall survival.^7^

In addition, consumer awareness is steadily changing. Social media platforms enable consumers to connect and gain information about the latest treatments and clinical trials nationally or internationally.^8,9^

These advances may translate to patients contemplating travel, including air travel, for medical reasons, such as for second opinions, for treatment at their cancer center of choice, or to explore trial opportunities. Others may do so for personal reasons. In some cultures, it is meaningful for patients to die in their homeland, necessitating travel in the (pre)terminal phase.

For the oncology multidisciplinary team members providing care, the risk assessment of air travel in particular is a complex problem posed in the daily clinical setting. Factors to consider include, but are not limited to, the patient’s physical capabilities, the distances involved, and the country of destination.^10^ Although commercial airlines routinely specify that anyone with a terminal or serious illness requires medical clearance^11,12^ there is a paucity of data or guidelines for clinicians to certify fitness to fly. We present the challenges of traveling with cancer, with a specific focus on air travel and a review of the scientific literature, which we examined to critically evaluate the current data on this topic.

**Context**

**Key Objective**
Do oncologists have data to guide them in assessing whether patients with cancer are fit to fly? The current evidence on traveling with cancer, with a specific focus on air travel, was collated, critically evaluated, and summarized in a narrative review.
**Knowledge Generated**
Only sixteen published articles were found relevant to traveling among patients with cancer, with no specific criteria found to guide the oncology community in assessing the risks of air travel and no randomized studies performed. However, the British Thoracic Society and Aerospace Medical Association Medical Guidelines had useful recommendations for fitness to fly among patients with cancer.
**Relevance**
In the absence of high-quality studies, individual fitness to travel should be assessed on a case-by-case basis, using existing recommendations as a guide.

## SEARCH METHODS

A review using Preferred Reporting Items for Systematic Reviews and Meta-analyses guidelines was performed. A search using the terms “cancer,” “advanced cancer,” “metastases,” “brain edema,” “lymphoedema,” “pneumothorax,” “pleural effusion,” “pericardial effusion,” “pneumonitis,” “hypoxia,” “end-of-life,” and “shunt,” combined with “flying” and “air travel,” was conducted. These search terms were selected because they were conditions most likely to be associated with malignancy and its treatment. The PubMed and Cochrane databases were searched for English-language studies up to December 2018. The retrieved studies were screened and reviewed for relevance. Studies, case reports, or guidelines referring to travel in the context of adult patients with malignancies were included. Airline and transport organization guidelines were hand searched and reviewed for relevance. A total of 745 published articles were identified; 16 studies were included. Because of the paucity of literature, we used an inclusive approach to data extraction. All eligible data were included to avoid omitting findings of potential value.

## RESULTS

Sixteen published articles were found relevant to our topic. We graded the studies according to the grading system listed in Table 1. We categorized the findings from these studies into pretravel, during travel, and post-travel issues. Table 2 lists the studies ranked on the basis of the level of evidence.

**TABLE 1:**
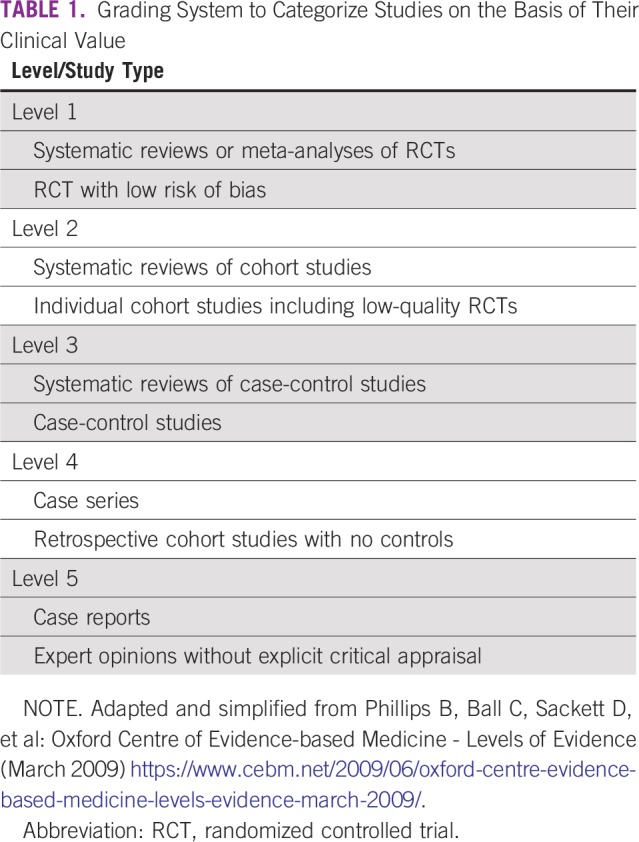
Grading System to Categorize Studies on the Basis of Their Clinical Value

**TABLE 2:**
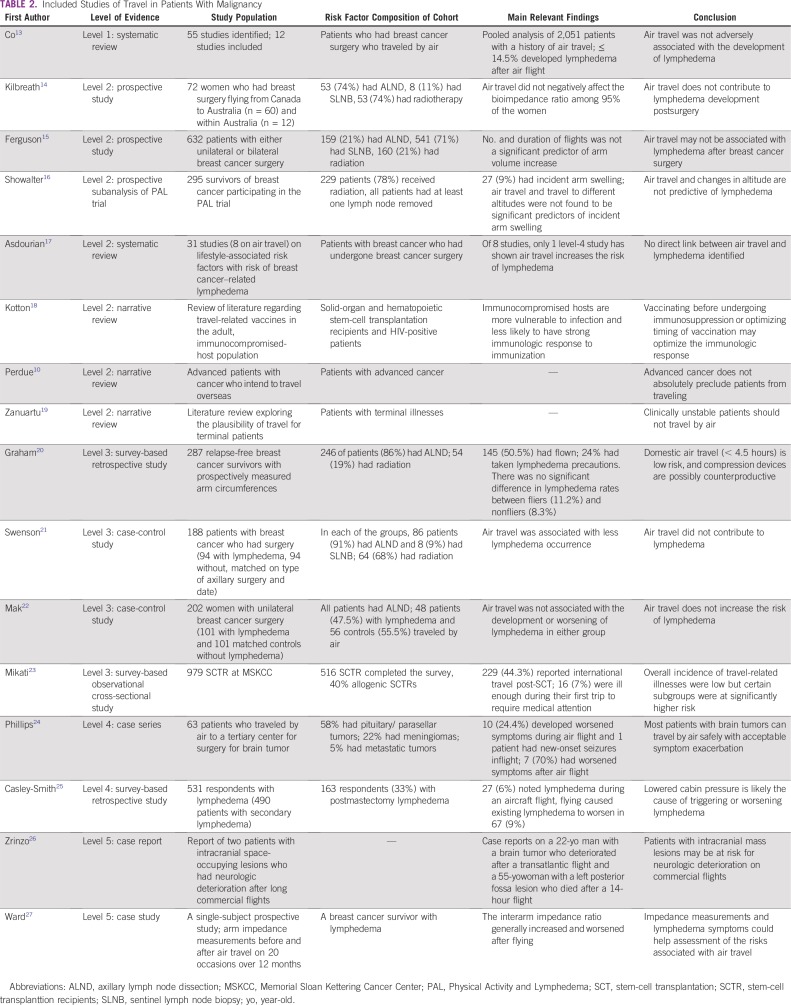
Included Studies of Travel in Patients With Malignancy

### “Doctor, Am I Fit to Fly?”

There are no specific criteria to deem a patient with cancer fit to fly. As a general rule, patients who are clinically unstable, currently receiving intensive radiation or systemic treatment protocols, or are terminal should not attempt commercial air travel.^4^ Clinical instability combined with the stresses of flight could pose a serious threat to the patient,^4^ not to mention the difficulty in obtaining medical care or emergent medical interventions midflight.

#### Neurologic implications.

##### Patients with primary brain tumors or brain metastases.

The emergence of local and more effective systemic treatments has rendered brain metastases as one of the new frontiers in cancer survivorship.^28,29^ Patients with treated brain metastases may not only enjoy a good quality of life, but also have new opportunities to participate in clinical trials in select tumor streams.^29^ Hence, it is not uncommon for patients with known cerebral disease to plan travels, including overseas.

There are no specific airline policies or regulations regarding patients with primary brain tumors or cerebral metastases. The effects of air travel on intracranial pressure warrant additional investigation,^30^ but studies in such circumstances are difficult to perform. The biggest study published was a case series of 63 patients with brain tumors traveling via commercial airlines for surgery. The authors found that most patients with brain and skull base tumors can travel safely via commercial airlines with acceptable symptom exacerbation. However, they cautioned that corticosteroids and anticonvulsants should be considered in patients who are symptomatic or have relatively large tumors with mass effect and peritumoral edema.^24^ Although cerebral disease should not be affected by reduced pressure, moderate hypoxemia at a high altitude could theoretically lower an already reduced seizure threshold.^31^ Oxygen delivery may be decreased in patients who are elderly, are volume depleted, are anemic, or have significant cardiopulmonary disease.^4^ Patients with seizures should also be made aware of other potential seizure-lowering threshold effects of fatigue, delayed meals, alcohol intake, or disturbed circadian rhythm on long flights.^4^

##### Recent neurosurgery.

There is a lack of evidence on when a patient can fly after neurosurgery.^32^ Any neurosurgical procedure may cause seizures, ischemia, and inflammation.^33^ Endoscopic intracranial surgery has less retraction injury to the adjacent normal brain, but all the risks of open surgery are still present, although relatively less.^32^ If intracranial air is present postcraniotomy, it will be reabsorbed over weeks. Gas trapped within the skull will cause increased intracranial pressure when it expands at high altitudes, but it is not known at which level of pneumocephalus and at which starting intracranial pressure complications would develop.^34^ Patients should have a computed tomography head scan for evidence that any intracranial gas has been absorbed.^4^ Flying should be considered contraindicated if there is any residual air within the cranial cavity.^11,12^ Cerebral spinal fluid leaks from any cause also raise the possibility of backflow and microbial contamination.^4^

#### Cardiothoracic implications.

##### Respiratory symptoms.

Patients who have undergone a recent pneumonectomy or lobectomy have reduced pulmonary reserve that may only become apparent during flight.^4^ The British Thoracic Society guidelines suggest that patients with significant respiratory symptoms, comorbidities exacerbated by hypoxemia, recent pneumothorax, risk of or previous venous thromboembolism, and a preexisting requirement for oxygen should be assessed for fitness to fly, including referral to a respiratory physician and possibly a hypoxic challenge test.^31^ The purpose of a hypoxic challenge test is to determine the need for inflight oxygen by exposing the patient to the hypoxia experienced at a cabin altitude of 8,000 feet while measuring hypoxemia and assessing symptoms. This is simulated using a mixture containing 15% oxygen, which the patient breathes for 20 minutes. If the arterial oxygen pressure is less than 6.6 kPa (< 50 mmHg) or blood oxygen saturation is less than 85%, inflight oxygen is required.^35^

Patients with a usual oxygen requirement flow rate exceeding 4 L/min at sea level will experience respiratory decompensation inflight. Hence, air travel is contraindicated.^31^ Those with symptomatic lymphangitis carcinomatosa, especially if the patient’s arterial oxygen pressure and respiratory function are compromised, or superior vena cava obstruction should only fly if absolutely essential, and they should have inflight oxygen available.^31^ Patients with major hemoptysis are at risk for exacerbation and should be cautioned against flying.^31^

Large pleural effusions should be drained at least 14 days before the flight, with post-thoracocentesis chest imaging to assess pleural fluid reaccumulation or for pneumothorax.^4^ It is recommended that patients with a current closed pneumothorax should not travel on a commercial aircraft, whereas those with a previous pneumothorax will need a chest x-ray confirming resolution before traveling.^31^

##### Malignant pericardial effusion.

No literature was found regarding flying in the setting of a malignant pericardial effusion. Theoretical concerns of cardiac tamponade and circulatory collapse should be considered in the context of the size of the pericardial effusion and the patient’s malignancy.

#### Implications of abdominal surgery and abdominal complications.

Aerospace Medical Association guidelines recommend delaying air travel for 7 to 14 days after major surgical procedures.^4^ Because of the occurrence of relative ileus for several days postsurgery, there is an increased risk of suture line tears, bleeding, and perforation.^4^. Laparoscopic abdominal surgical procedures are less associated with ileus than open procedures and are not as restrictive. Flights can occur the next day if bloating symptoms are absent.^4^ Patients with bowel obstruction or diverticulitis are advised to wait 7 to 10 days after resolution before air travel.^4^

#### Implications of postoperative lymphedema.

The precipitating factors for lymphedema postsurgery/postirradiation are uncertain, but may include pressure changes resulting from airplane travel.^27^ Only a single study has shown a significant correlation between air travel and lymphedema in a small number of patients.^25^ Although one study reported that air travel of less than a 4.5-hour duration represented a low risk for lymphedema,^20^ others reported that air travel did not increase lymphedema risk.^14-16,21,22^ A systematic review found that air travel was not adversely associated with the development of lymphedema after breast cancer surgery.^13^

#### Implications of thromboembolism.

In the setting of a deep vein thrombosis (DVT) or pulmonary embolism, airlines mandate medical clearance within 21 days of the event, and patients need to be stable while taking anticoagulants, with normal respiratory function before being allowed to fly.^11,12^ In fact, it is recommended that patients who have had a recent DVT should not fly for 4 weeks or until the DVT has been treated and there is no evidence of pre-exercise or postexercise desaturation.^31^

#### Implications of travel in the context of clinical trials.

Meticulous planning is required if patients require travel while participating in a clinical trial. Organizing travel logistics, such as cost, medication supply, monitoring adverse effects, and ensuring strict adherence to treatment protocol, will vary.

### “What Do I Need to Prepare Before I Go?”

#### Medications and vaccinations.

An adequate supply of oral cancer medications, as well as supportive medications such as antinausea, antidiarrheal, analgesic, and other medications that might help with symptom control, will need to be provided to patients. Restrictions on the quantity of medications such as opioids and supportive documentation should also be considered, particularly when visiting countries with different legal consideration for the possession of restricted medications. When traveling to different time zones, also consider that disruption in daily routine can cause confusion concerning timing of medications.^5^

Travel physician advice on vaccinations on the basis of the intended travel destination can be helpful because the immunocompromised host is less responsive to vaccinations, and protective levels of vaccines may be of shorter duration. Studies are lacking to evaluate the response to travel-related vaccines in immunocompromised patients with cancer or stem-cell transplantation recipients.^18,23^ Thus, specific guidelines for these groups are absent.^23^ Complete recovery of the immune system may take up to a year in patients treated with lymphocyte-depleting agents, thus increasing the risk of opportunistic infections and precluding the use of live vaccines.^23^. To optimize the immunologic response, immunocompromised hosts should be vaccinated during periods of no or low exogenous immunosuppression when possible.^18^ Vaccination for travel should be started several months before the trip to allow time for serologic evaluation with possible additional boosters.^18^

#### Biochemistry.

There were no published data to indicate the minimum hemoglobin level at which it would be safe to fly, but major airlines recommend a level of 8.5 g/dL or more.^31^ Electrolyte imbalances should be corrected where possible and travel avoided if the patient is symptomatic or unstable.^31^

#### Respiratory function.

Sea-level oxygen saturation poorly identifies those at risk who will desaturate to below 90% during routine commercial flights.^37^ One third of patients with a sea-level blood oxygen saturation of 92% to 95% but no other risk factor desaturated below 90% during a hypoxic challenge.^37^ However, hypoxic challenge might not be necessary in patients whose oxygen saturation is at or above 95%. No consensus exists regarding assessment methods or criteria for recommending oxygen.^38^ A hypoxic challenge test could assist in determining suitability for flight and need for supplemental oxygen inflight. If supplemental oxygen is required, medical clearance is essential.^11^

#### Venous thromboembolism.

Patients with active malignancy are considered at high risk for venous thromboembolism (VTE), and the risk of VTE is greatest on flights lasting more than 8 hours.^31^ A Cochrane review found that airline passengers could expect a substantial reduction in the incidence of symptomless DVT if they wore compression stockings on flights longer than 5 hours.^41^ A preflight prophylaxis dose of low molecular weight heparin for both outbound and inbound journeys should be considered, in addition to other general recommendations, such as avoiding excess alcohol and caffeine-containing drinks, maintaining adequate hydration during the flight, remaining mobile, performing inflight exercises wearing compression stockings, and avoiding the use of sedatives.^31^ Aspirin alone is not recommended.^52^

#### Postoperative lymphedema.

No consensus exists with reference to the risk conferred by air travel on the development of lymphedema and the utility of compression garments as prophylaxis on flights.^17^ One study called into doubt the safety and efficacy of compression garments and noted that should a compression garment be worn, it is to be checked carefully near the time of the flight for the correct fit.^20^ The position statement of the National Lymphedema Network recommends the use of compression garments during air travel for people with a confirmed diagnosis of lymphedema.^40^ Specifically for breast cancer, there is no evidence to show that prophylactic compression sleeve use is or is not of benefit^17^.

#### Physical logistics.

A considerable amount of physical activity is also involved in travel, much of it carrying, pushing, or pulling bags, which may well be in excess of the passenger’s normal exercise limits. Most airports provide excellent services to assist the disabled passenger, and arrangements should be made beforehand, if possible.^5^

#### Medical clearance.

Medical clearance forms vary among airlines, but information is generally available on the respective airline Web sites. Commonly, medical clearance is required when traveling within a certain period of time after a medical event.^11,12^

#### Travel insurance.

For international travel, some travel insurers may cover cancer-related costs in select circumstances, for example, prolonged periods of controlled disease or not having required treatment within a certain time period. However, most patients with active cancer or receiving treatment will find it difficult to obtain any travel health insurance cover. Even if provided, the patient may be charged an assessment fee or a higher premium.^42^ Patients need to be made aware of out-of-pocket expenses in the setting of unexpected hospital presentations, emergency procedures, and need for repatriation because often these costs may be high, depending on the countries involved. The level of medical capability will also vary among countries, and patients should consider what, if any, care they may require when abroad and whether these needs can be met, should they require it.

#### Other documents.

A written summary of the patient’s cancer treatment should be provided by the physician to the patient, including diagnosis, treatment, and contact details of treating physicians and family members. It would also be useful to have results of prior investigations, such as chest x-rays, computed tomography scans, or other abnormal tests. This information should ideally also be translated into the language of the country of destination.^10^ Patients should also ensure that their wills and advanced health directives are updated.

### “What Kind of Problems Can I Get Into During the Flight?”

#### Air cabin pressures.

Although modern aircraft cabins are pressurized, cabin air pressure at cruising altitude is lower than air pressure at sea level. As airplanes ascend, the cabin pressure is maintained at a level that corresponds to the outside air pressure at 6,000 to 8,000 feet above sea level, depending on the route and type of aircraft. This correlates to oxygen concentrations of approximately 15.1% to 17.1%,^43^ which results in an estimated blood oxygen saturation of 90%.^4^ Blood oxygen levels may drop by an average of 5% at cruising altitudes on both short- and long-haul flights.^44^ Newer aircrafts, such as the Boeing Dreamliner, are able to maintain cabin air pressure at not more than 6,000 feet, which correlates to a smaller drop in blood oxygen levels.

Cabin air undergoes a degree of recycling, as well as exchange with atmospheric air. This process leads to increased inspired fraction of carbon dioxide levels in aircraft cabins during flight. A mild degree of hypercapnia can lead to cerebral vasodilation,^43^ which in turn raises intracranial pressure.^44^ Furthermore, expansion of gas at lower barometric pressure may cause cerebral edema, which could also exacerbate any increase in intracranial pressure. Exposure to long commercial flights possibly leads to mild cerebral hypoxia and edema similar to that in acute mountain sickness and high-altitude cerebral edema. Decompensation as such can further elevate preexisting increased intracranial pressure.^45^

There were no published data on the role of prophylactic corticosteroids preflight to prevent development or exacerbation of edema in patients with cerebral tumors, except for the study by Phillips et al^24^ noting an inverse correlation between periflight corticosteroid usage and symptom exacerbation. There were also no published data concerning the influence of altitude on the frequency of intracranial hemorrhage.

Inflight acute mountain sickness and life-threatening high-altitude cerebral edema at 11,800 feet altitude have been reported after head and neck surgery or radiation, thought to be the result of a lack of hypoxic ventilatory response from dysfunctioning carotid bodies. Prior hypoxic ventilatory response testing may be useful in this group.^47^

In flight, gas in body cavities will expand up to 30%. In intestinal obstruction, a flare in the patient’s symptoms; including nausea, abdominal distention, and pain is expected.^19^ For patients with colostomies, intestinal distention may increase fecal output.^4^

#### Air cabin humidity and risk of airborne disease transmission.

Aircrafts also have low cabin humidity, usually ranging from 10% to 20%.^4^ This could aggravate thick secretions in patients with tracheostomies, laryngectomies, vocal cord paralysis, or laryngeal dysfunction. Humidified oxygen, adequate hydration, and suctioning can reverse some of these effects.^4^

The risk of airborne disease transmission within the confined space of the aircraft cabin is difficult to determine. Commercial airlines are a suitable environment for the spread of pathogens. Transmission of infectious diseases probably happens more frequently than reported for various reasons, including reporting bias and the fact that most diseases have a longer incubation period than air travel.^48^ Patients receiving chemotherapy should be aware of peak timing of neutropenia and the risk of infection, and potentially avoid traveling during those periods wherever possible.^31^

#### Medical devices.

Gas expansion also affects medical devices, such as pneumatic splints, feeding tubes, urinary catheters, and cuffed endotracheal or tracheostomy tubes. Gas-expansion concerns in these devices can be eliminated by instillation of water rather than air during air travel.^49,50^ There were no published data on the effects of reduced atmospheric pressure on the dynamics of pump mechanism, such as continuous subcutaneous infusions of medications through a battery-powered syringe driver, but they should not preclude air travel.^31^

#### Resuscitation orders.

An aircraft in midflight is a unique environment in which to provide medical care.^3^ Although advanced health directives or acute resuscitation plans may exist, airline crew members are not mandated by law to follow these directives during inflight emergencies. Therefore, patients and their surrogates should know that cardiopulmonary resuscitation may be initiated.^51^

### “What Problems Could Happen When I Get There?”

Patients and carers should be aware of potential unforeseen expenses related to illness once they arrive at their destination. These include repatriation, medical escorts, or even air ambulance. In the event of death, arrangements to repatriate the mortal remains back can be challenging and expensive.

## DISCUSSION

The number of patients with cancer who are undertaking air travel is growing every year as a result of lower costs and increasing ease of air travel but also improvements in therapies resulting in better patient performance status and survival. Yet, we found a paucity of evidence to guide the oncology community in assessing the risks of air travel for these patients. There were no specific criteria found and no randomized controlled studies performed.

The case series by Phillips et al^24^ was the biggest study performed thus far in patients with brain tumors. The authors found that patients with completely asymptomatic tumors did not develop any symptoms during flight, whereas there was an inverse correlation between periflight corticosteroid use and symptom exacerbation. Their findings support the common clinical practice of prescribing corticosteroids to patients who are symptomatic from mass effect or peritumoral edema, and oncologists would especially do so for those who are planning to travel by air. In fact, many clinicians would not recommend traveling to those patients who were symptomatic.

For patients with lung cancer or lung metastases, or who have been through thoracic procedures, the guidelines suggest referring to a respiratory physician and possibly arranging a hypoxic challenge test. This is not always possible, especially for patients in rural or remote regions. The waiting time for a consultation with a respiratory physician is also sometimes a barrier because many patients do not give their treating team much time for assessment before their planned flying date. In these cases, clinical assessment is of utmost importance. Physical presentations such as preexisting respiratory symptoms, overall functional status, oxygen saturation at sea level, and risks of complications, such as comorbidities exacerbated by hypoxemia, recent surgery or procedures, and recent pneumothorax or pulmonary embolism, need to be taken into account.

Unsurprisingly, no literature was found regarding flying in the setting of malignant pericardial effusion. The condition is relatively rare and often goes undetected if asymptomatic, whereas symptomatic patients are usually less apt to travel.

Traditionally, both health professionals and patients alike had fears that flying would induce or exacerbate postoperative lymphedema. Of all the travel-related complications in patients with malignancy, this issue was the most well studied. Most of the studies were retrospective and observational. The vast majority of studies concluded that there is no evidence to restrict air travel because of risk of lymphedema. These findings are reassuring to patients who are often anxious about flying after breast cancer surgery.

Patients with malignancies are known to have a higher risk of venous thromboembolism while traveling. There is no solid evidence, but the British Thoracic Society recommends considering a preflight prophylaxis dose of low molecular weight heparin in addition to other general recommendations to reduce the risk of VTE while traveling. With new direct oral anticoagulants on the market and emerging studies of usage of direct oral anticoagulants in the cancer population, these recommendations could potentially change to include using these medications for VTE prophylaxis.

In confirmed venous thromboembolism, in the absence of clear evidence, patients should be assessed individually. As long as the respiratory function is adequate and the increased risk of bleeding related to anticoagulation is deemed acceptable, the patient should be able to travel on a commercial flight.

With regard to cardiopulmonary resuscitation, it is unfortunate that there are controversies regarding airline crew members not following advance health directives not to resuscitate during inflight emergencies because of airline regulations. There is a clear need for the development of guidelines to help patients, their carers, and physicians in the setting of air travel. The medical community may need to engage the airline industry to reach clarity about the safety of patients with cancer during air travel. Respecting patients’ wishes should be important at all times, regardless of whether they are in the hospital or midflight.

In conclusion, discussing safety to travel or fitness to fly is a necessary conversation between oncologists and their patients. Minimizing the potential risks can be a complex task, but careful planning and good communication can curtail unexpected obstacles. Valuable information may be sought from travel physicians, airlines, and the embassies or high commissions of destination countries. In the absence of large prospective studies, individual fitness to fly should be assessed on a case-by-case basis bearing in mind that maximizing a patient’s ability to safely travel is an important goal for many individuals with cancer.
